# PyOmeroUpload: A Python toolkit for uploading images and metadata to OMERO

**DOI:** 10.12688/wellcomeopenres.15853.2

**Published:** 2020-08-26

**Authors:** Johnny Hay, Eilidh Troup, Ivan Clark, Julian Pietsch, Tomasz Zieliński, Andrew Millar

**Affiliations:** 1 EPCC, University of Edinburgh, Edinburgh, EH9 3FD, UK; 2 SynthSys and School of Biological Sciences, University of Edinburgh, Edinburgh, EH9 3FD, UK

**Keywords:** Data sharing, research data management, microscopy, OMERO, metadata, Docker

## Abstract

Tools and software that automate repetitive tasks, such as metadata extraction and deposition to data repositories, are essential for researchers to share Open Data, routinely. For research that generates microscopy image data, OMERO is an ideal platform for storage, annotation and publication according to open research principles. We present *PyOmeroUpload*, a Python toolkit for automatically extracting metadata from experiment logs and text files, processing images and uploading these payloads to OMERO servers to create fully annotated, multidimensional datasets. The toolkit comes packaged in portable, platform-independent Docker images that enable users to deploy and run the utilities easily, regardless of Operating System constraints. A selection of use cases is provided, illustrating the primary capabilities and flexibility offered with the toolkit, along with a discussion of limitations and potential future extensions. *PyOmeroUpload* is available from: https://github.com/SynthSys/pyOmeroUpload.

## Introduction

### Background

Creating Open Data through sharing, discovery and re-use of research data are integral activities for promoting Open Science ^1^. Effective data management, storage and cataloguing strategies are essential for enabling open research of this sort, and are therefore vital activities for fulfilling the requirements of publicly- and charity-funded research, as re-stated for example in the BEIS Open Research Data Task Force report (2018) and elsewhere ^2– 4^. Workflows that include regular data sharing can support these activities. To that end, it is imperative that researchers are empowered with open tools, software and user communities that embed Open Science within ongoing research, not as an afterthought. We present an open source software toolkit designed to aid researchers by facilitating the management of imaging data and accompanying metadata.

Contemporary research in cell biology generates substantial volumes of microscopy data. The volume and velocity of data produced presents challenges for smaller laboratories that typically do not have the infrastructure, software or expertise to sustain bespoke resources and workflows for this data management task. Data discovery and re-use depend on high-quality metadata, including detailed descriptions of experimental conditions, materials used, operational procedures and analysis methods ^5^. Software intended to support this process must therefore encourage rich metadata definition. The more streamlined the process of depositing data, and enriching data with metadata that has been captured at the point of generation, the greater the quantity and quality of data that can be shared.

The foremost open source software platform for managing microscopy image data is Open Microscopy Environment’s (OME) Remote Objects (OMERO) ^6, 7^. OMERO is intended as a complete platform for managing images in a secure central repository where data can be viewed, organized, analyzed and shared online ^8^. The platform is frequently updated and supports importing over 150 image formats, full multi-dimensional image viewing, analysis with scripts and plugins, data conversion and publishing through URLs. OMERO also provides excellent cataloguing capabilities, where data can be annotated with tags, comments, key-value pairs, tables and supplementary files; images can be browsed or searched through accordingly, and shared with collaborators. Another excellent feature of OMERO is that it provides comprehensive support for a variety of users and software developers in the form of programming APIs for Java, Python, MATLAB and C++. Additionally, OMERO is supported by a very active community of microscopy researchers, while the OME staff runs regular workshops and engages very effectively with the online community via support forums.

### Rationale

One of the greatest barriers to wide adoption of open research principles and data sharing is the effort required to deposit data and accompanying metadata into an online repository. Hence solutions that can streamline and automate this process for researchers are needed. One of the research groups within our centre routinely perform time-lapse microscopy experiments in which organisms are monitored over a period of hours, with bright-field and fluorescence images captured every 2.5 minutes (for example Granados *et al.*, 2018) ^9^. The images are acquired using three optical channels across twenty or more latitudinal/longitudinal positions, through multiple z-planes. These kinds of data benefit hugely from the multi-dimensional ‘hypercube’ format feature in OMERO, that allows one single “OMERO image” to represent the whole recorded timeseries in a five-dimensional structure, including space, time and channel ^8^. At the time of initiating the experiment, the biological context is known (including strains, medium, and conditions) and it is the optimum moment to capture this information, for example in a text file. These types of experiments are perfect candidates for automation of data deposition, wherein large quantities of images are generated (typically 90,000 raster images or more, constituting 30 GB per experiment) and detailed descriptions can be constructed by combining technical metadata obtained from the experimental setup (such as time resolution, exposure time, z-positions) and user input.

Python has become a language of choice for biological applications, so easy integration of image data processing in this environment is welcome. At the same time, using Python software on Windows platforms – which dominate laboratories’ IT infrastructure and microscopy management software – can still be cumbersome.

A Python-based tool that facilitates microscopy data deposition was conceived: *PyOmeroUpload* toolkit, by which the data generated by laboratories’ microscopes could be programmatically uploaded to an OMERO server along with experimental metadata, thus removing the burden on researchers of manually performing this process. *PyOmeroUpload* performs two principal functions: it parses metadata from user-friendly, human-readable semi-structured text files and creates multi-dimensional images from a directory structure populated with multiple images across multiple dimensions, before uploading the reconstituted data and extracted metadata to an OMERO server. For heavy data producers, this drastically reduces the resource cost of uploading and cataloguing their data and offers the additional benefit of enforcing lab-specific metadata conventions.

*PyOmeroUpload* complements the presently available tools for deposition of data and metadata in OMERO: *OMERO.insight* ^10^ and *OMERO.cli* ^11^. The Insight client is a desktop application featuring a rich GUI (Graphical User Interface) for viewing and importing data, using the Bio-Formats ^12^ library for translation of proprietary file formats. The CLI is “a set of Python-based system administration, deployment and advanced user tools” ^13^ that allows users to import images to an OMERO server from the command line, typically via a Shell script. Neither of these is capable of transforming raw two-dimensional image data into multi-dimensional ‘hypercube’ images, and neither offers a method of automatic metadata extraction.

The *PyOmeroUpload* library permits easier interaction with the OMERO server than with the standard API by presenting a collection of higher-level functions that simplify session management, ad-hoc uploading and querying. Newer releases (>= 5.6) of the Python OMERO library ( *omero-py*) ^14^ are distributed through PyPi ^15^ and Conda ^16^, which support Windows, Linux and Mac OS Operating Systems (OS). Previous versions (< 5.5) of the Python OMERO library were distributed through the Conda Bioconda ^17^ channel, which only supports 64-bit Linux and Mac OS systems. Since many biology research labs use Windows OS, this constraint presented a significant obstacle.

To address these issues we prepared pre-packaged, virtualized *Docker* ^18^ containers that allow easy use of *PyOmeroUpload* by both Windows and Linux users directly through the command line, Python Shell, in code or via *Jupyter* Notebooks ^19^. Our software was developed during the period that OMERO continued to be built with Python 2 and distributed through Bioconda, so using the client Python libraries was challenging on Windows systems, and the Docker solution provided a viable option for Windows users. Although recent releases of omero-py can be installed through Conda or Pip on all OSs, our fully portable, packaged toolkit offers further convenience for users since these containers also have the advantage of hiding any systems administration activities required for accessing OMERO from within Python from less experienced users.

## Implementation

### PyOmeroUpload

The PyOmeroUpload toolkit software architecture comprises three main components: the metadata parser, data transfer manager and data broker. The data transfer manager provides a high-level interface for transferring data in a specified directory structure to a remote OMERO server. The data broker service makes extensive use of the OMERO Python API modules, exposing core functions for administering HTTP sessions, creating OMERO datasets and multi-dimensional images, and linking metadata objects. The uploader software is extensible, since the metadata parser can be replaced if required for a particular use case. Likewise, the image processing can be performed by a custom code module to compose the data into the multi-dimensional images.

The OMERO Python API is the core dependency for connecting to and managing sessions with the OMERO server, as well as for retrieving data objects and executing queries. The OMERO API can be used with the Blitz Gateway, “a Python client-side library that facilitates working with the OMERO API” ^20^, or it can be utilized via a number of lower ‘service’ levels that provide stateless access. Although accessing the OMERO Python API with the Blitz Gateway as a context manager is encouraged, we found the stateless service level APIs to be more powerful and flexible.

### OMEROConnect

To enable the software for Windows OS users, and to minimize complexity, the OMERO Python library and PyOmeroUpload package are wrapped into portable Docker image definitions provided in the OMEROConnect repository. These images are specified by a hierarchy of Dockerfiles that build upon one another, inheriting from a base image which incorporates all the necessary libraries for OMERO access. There are four Docker images in total, as described in Table 1.

**Table 1.** OMEROConnect Docker images.

Image Name	Description	Parent Image	Docker Pull Command
omero_base	The base image that contains environment necessary for the OMERO Python API libraries	openjdk/11 ^22^	docker pull biordm/omero- connect:omero_base
omero_ uploader	Image with the installed PyOmeroUpload library	omero_base	docker pull biordm/omero- connect:omero_uploader
omero_jupyter	Image that contains Jupyter Notebook server with OMERO API, PyOmeroUpload and common scientific libraries	omero_uploader	docker pull biordm/omero- connect:omero_jupyter
omero_ide	Container with fully-fledged graphical IDE for python development	omero_jupyter	docker pull biordm/omero- connect:omero_ide

## Operation

### Installation – Conda

Basic installation on Windows, Linux and Mac OS systems is by Conda, following the typical usage pattern. The OMERO Python library requires Python 3.6 or greater, so a corresponding Conda environment can be created. The commands ^14^ are as follows:


$ conda create -n omero_upload -c ome python=3.6 zeroc-ice36-python omero-py
$ conda activate omero_upload
$ pip install git+https://github.com/SynthSys/pyOmeroUpload.git@v5.6.2_2.0.0


Alternatively, the provided Docker containers can be used instead, as described in the section below.

### Installation – Docker

For an alternative installation using pre-built Docker images, which work regardless of host OS or available system libraries, Docker containers can be deployed (and the instructions at https://docs.docker.com/docker-for-windows/install/ can be followed to install Docker for Windows). The relevant Docker images can be pulled from DockerHub ^21^ with the commands listed in the table above.

For example, to utilize the library from the command line, the following commands can be executed (for downloading the Docker image, starting the Docker container, connecting to it and accessing the uploader library):


$ docker pull biordm/omero-connect:omero_uploader
$ docker run -t -d --name omero-uploader --entrypoint /bin/bash biordm/omero-connect:omero_uploader


In order to access local files, the docker volume should be mapped to a local folder using the -v command option. For instance, to access data in C:\Temp\omero_data, the command is as follows (where the local mounted files will be available in the ~/work directory from within the container):


$ docker run -t -d --name omero-uploader -v 
'/c/Temp/omero_data:/home/jovyan/work:rw' --entrypoint /bin/bash biordm/omero-
connect:omero_uploader


**Hint**: Windows users should enable access to the local drive in the Docker Desktop app settings (under Settings » Resources » File Sharing).

**Hint**: Before running a Docker container with new parameters, the old one must be removed first, or the new one can be labelled with a different name to avoid conflicts.

**Hint**: Some useful commands for listing all containers, stopping and removing a container are as follows:


$ docker ps -a
$ docker stop {CONTAINER_ID}
$ docker rm {CONTAINER_ID}


### Using the toolkit

The main entry point is the function launch_upload in the PyOmeroUploader class of the pyomero_upload module. The function accepts parameters for creating a connection to the desired remote OMERO server and for defining the location of metadata and image files. For example, the following commands can be executed from within the Python Shell (Windows users must run this from within the Docker container, after the `docker exec…` command):


$ python
>>> from pyomero_upload.pyomero_upload import PyOmeroUploader
>>> uploader = PyOmeroUploader('USERNAME', 'PASSWORD', 'demo.openmicroscopy.org')
>>> uploader.launch_upload('dataset_name', '/path/to/data', True)


## Use cases

The toolkit has been designed with a range of researchers, data generators, curators and developers in mind so a variety of use cases can be explored.

To demonstrate the use cases below, we use the Docker images, as they work on both Linux-like and Windows systems, and provide test data to be used in the examples below. Follow the steps below (in Windows PowerShell) before exploring the use cases:


# retrieving test data

$ cd C:\Temp
$ git clone https://github.com/SynthSys/omero_connect_demo

# getting and starting Omero connect docker
$ docker pull biordm/omero-connect:omero_jupyter
$ docker run --name omero-jupyter -p 8888:8888 -v 
'/c/Temp/omero_connect_demo:/home/jovyan/work:rw' biordm/omero-connect:omero_jupyter



These commands download the demo Jupyter notebooks and data from the omero_connect_demo repository, then run a container named omero-jupyter which has a work directory linked to the downloaded demo data (at C:\Temp\omero_connect_demo).

**Figure 1.**
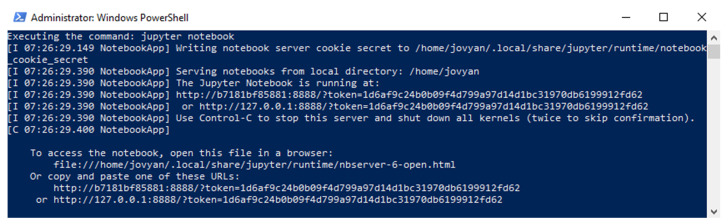
Windows PowerShell terminal showing output of the Docker run command for an omero_jupyter container.

The user should see output similar to that in Figure 1, where the localhost with exposed port URL and unique token are provided for accessing the Jupyter Notebook instance running in the Docker container. The user should make a note of the access token (the string starting with “token=”) as it is needed to access the notebooks, and leave this terminal open in the background with the running Docker container.

In the use cases below, the OMERO public demo server is the target instance; users must provide their own login credentials for access to this server, and this requires registration ^23^. You can register for a free demo account for OMERO at https://help.openmicroscopy.org/demo-server.html.

### Use case 1: uploading a data folder

The primary use case is for data generators and curators who wish to deposit their data in an OMERO server, typically as part of a data sharing workflow. PyOmeroUpload can be of particular benefit to these users because it supports the automation of such processes by including a CLI tool that is easily integrated with other programs or scripts.

We provided a simple script – upload_cli – that invokes the necessary Python functions to perform an upload, and it can be modified for individual needs. To upload the provided test data (mapped from the local file system in the steps above) into the OMERO demo server, run the following commands from a new shell window:


$ docker exec -it omero-jupyter bash
$ cd work
$ python -m pyomero_upload.upload_cli -d test_data -n my_first_dataset -u USER -s
demo.openmicroscopy.org -y


**Figure 2.**
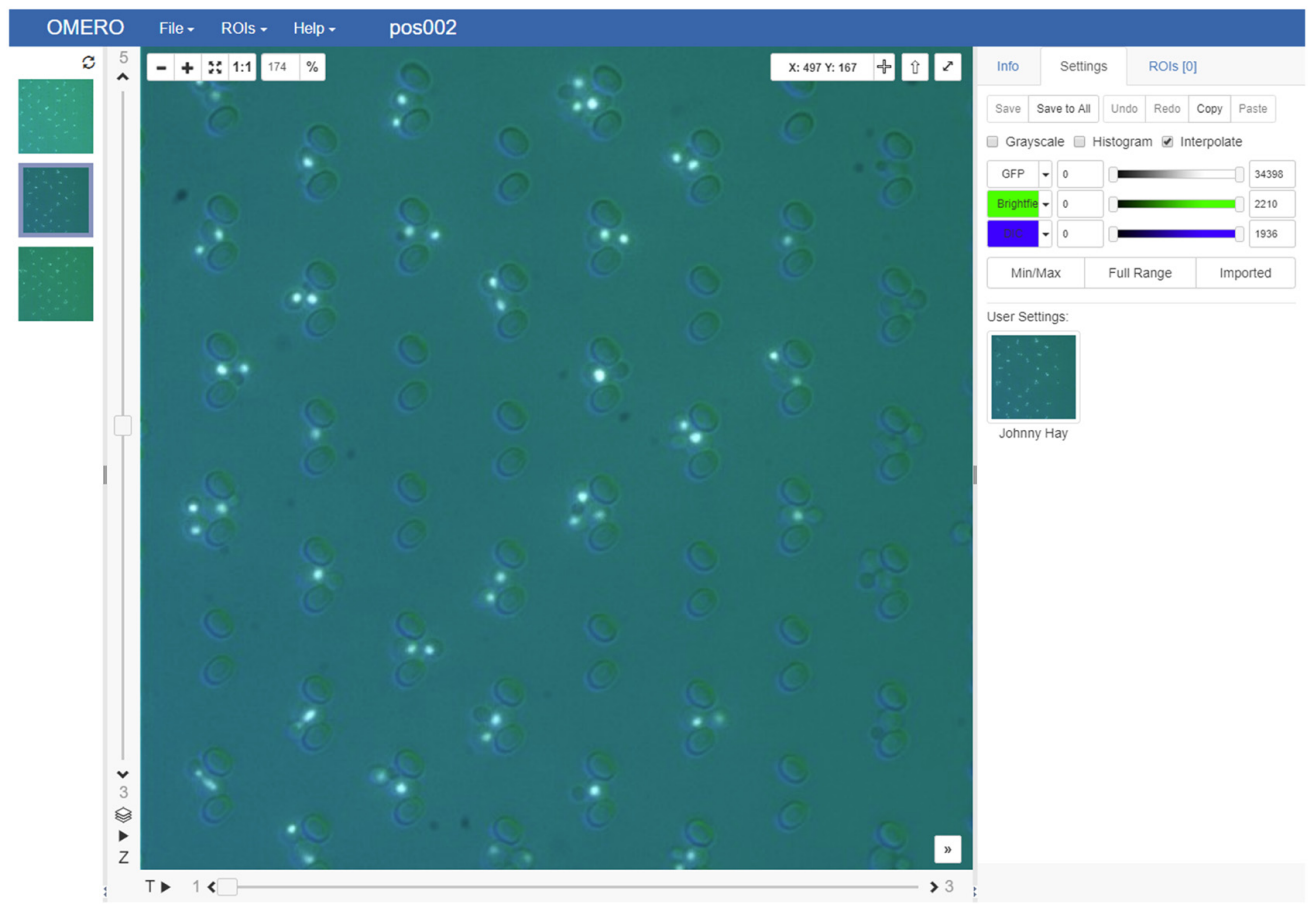
Screenshot of an uploaded hypercube image as displayed in the OMERO web client ‘iviewer’ GUI.

After completion, the script creates a new dataset in demo OMERO. The dataset consists of 3 cubes with additional dimensions for 3 channels and 3 timepoints as shown in Figure 2.

For more advanced use cases, the provided data transfer script can be invoked with additional parameters as described in the README under https://github.com/SynthSys/pyOmeroUpload/blob/master/README.md.

The provided, default implementation transforms images into five-dimensional hypercubes following the rules:

1. Target directory contains sub-directories named ‘pos{xxx}’, each of which corresponds to a microscope position, where ‘{xxx}’ is a unique numeric identifier for that position
2. Within each sub-directory, there are multiple image files per z-section, time point and channel
3. Each image file adheres to a naming convention of ‘{abc}_{timepoint}_{channel}_{z-section}’ where ‘{abc}’ can be any arbitrary string

**Figure 3.**
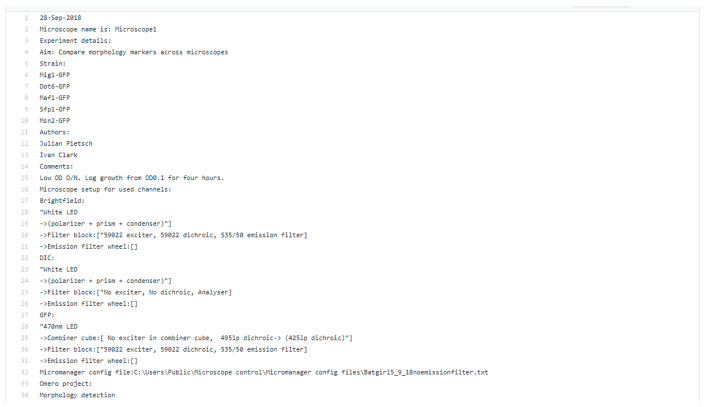
Text in the log.txt file that is parsed, and where KVPs are extracted from, by the default PyOmeroUpload metadata extractor.

**Figure 4.**
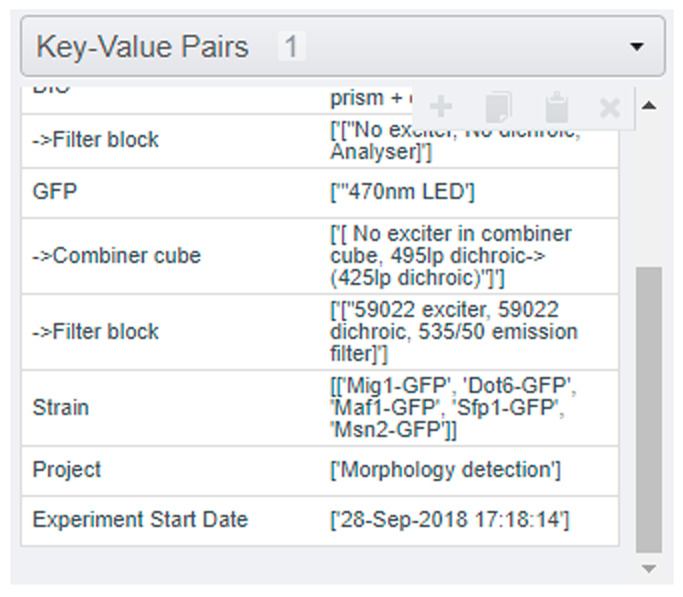
Screenshot of the KVPs as they appear in the OMERO server UI.

For insight into how the metadata is extracted by the default metadata parser, the ‘*Acq.txt’ and ‘*log.txt’ files can be inspected (in the test_data). For ease of reference, an excerpt of the beginning of the example ‘*log.txt’ file is shown in Figure 3.The metadata tags and key-value pairs in OMERO are generated from colon-delimited key-value pairs in the text files, while the tables are generated from tab-separated tabular text and attached to the dataset as ‘h5’ files. Examples of the KVPs are displayed below in Figure 4. Pre-defined regular expressions are used to extract particular elements of metadata, and these could be modified for individual needs.

### Use case 2: interactive OMERO operations with Jupyter

In the second use case, users can interact with OMERO through Jupyter Notebooks using the provided docker image.

Using the same Docker container as before in Use Case 1, users should pay attention to the startup screen as in Figure 1. When the Docker container is started for the first time, a unique token is displayed for accessing the Jupyter Notebook instance running within. The token must be provided either in the URL or on the login page to access the Jupyter Notebook, and if the Docker container is restarted then this token is not displayed again.

By visiting the address http://127.0.0.1:8888 in a browser, the Jupyter Notebook server can be accessed, and the notebooks shown in the table below are available in the ‘work’ directory (after logging in with the token). To begin with, the omero_upload notebook demonstrates invocation of the launch_upload entry point function with appropriate parameters. After running the upload cell is completed, the output will report the number of images deposited along with the destination dataset identifier. Once data are uploaded, the target server is queried in a successive cell to retrieve and verify the corresponding dataset. The query notebook provides more extensive (meta)data retrieval and exploration operations, while the API notebook utilizes the OMERO JSON API to retrieve metadata through an alternative mechanism (see Table 2).

**Table 2.** Demonstration Jupyter Notebooks.

Sample Notebook	Description	Features
omero_upload.ipynb	Demonstrates how to connect upload data by invoking the PyOmeroUpload	• Importing modules • Configuring OMERO server connection parameters • Uploading a data directory
omero_query.ipynb	Demonstrates how to query the (meta)data services using the standard OMERO Python library	• Using OMERO Python library client • Constructing and executing parameterized HQL queries • Exploring the OMERO object model and hierarchy
omero_api.ipynb	Demonstrates how to query the (meta)data services using the OMERO JSON API ^28^, without depending on the PyOmeroUpload client	• Using the Python ‘requests’ ^29^ library to connect with and query the OMERO JSON API over HTTP • Processing JSON responses

It is possible to use the notebooks on Linux-like platforms outside of the Docker container, after following the relevant installation steps for the pyOmeroUpload library.

These notebooks demonstrate interactions with OMERO, and can be adapted for deeper analysis and visualization using the preinstalled Python libraries such as for example *Pandas* ^24^, *NumPy* ^25^, *Matplotlib* ^26^ and *seaborn* ^27^. If other Python libraries are required, they can be installed with the following commands:


$ docker exec -it omero-jupyter bash
$ conda install {PACKAGE_NAME}



### Use case 3: implementing a custom metadata parser or image processing module

There are myriad file formats, structures and ontologies associated with metadata collected during experimental data generation. The captured metadata are stored either in semi-structured text or according to customized schema. It would be an impossible task to develop a software that could extract meaningful information from such a variety of inputs. Therefore, the PyOmeroUpload toolkit is designed with modularity in mind, allowing specification of a custom metadata parser and image processing module at runtime.

The custom metadata parser extends the abstract base class ‘MetadataParser’. The interface is simple and mandates only one function, ‘extract_metadata’, which must return a Python dictionary containing a description and any metadata tags, key-value pairs and table elements in the form of ‘{ “description”: “”, “tags”: [], “kvps”: [], “tables”: [] }’. The tags list is simply a collection of string values while the KVPs list is a nested array containing keys paired alongside their corresponding values within sub-arrays, and the tables list is a collection of Pandas *DataFrame* ^30^ objects, complete with name attributes that have been assigned a value. The dictionary object is then passed to the broker instance which processes and uploads the metadata as children objects linked with the parent dataset in the OMERO server. Our own implementation, ‘MetadataAggregator’ which combines extracted metadata from two further implementations – ‘AcqMetadataParser’ and ‘LogMetadataParser’ – can be used as an example of the parser. 

The structure of the metadata parsing component in PyOmeroUpload permits a great deal of flexibility for customisation. For example, users can easily create an alternative implementation that extracts imaging parameters from various custom acquisition programs, or makes use of the utilities provided by BioFormats for doing so. By capturing the technical microscopy metadata (for example channels and exposure times etc.) in addition to experimental metadata (for example strains, project aims and related research etc.) which preserves the biological context of the data, such custom parsers could help to enrich the data deposited in OMERO.

Customized classes for image processing should inherit from the image processor interface, and must implement the ‘process_image’ function which expects a reference to the current OMERO server session connection, a file path, and a reference to the target OMERO dataset object. The function should contain the logic required to pre-process for example to form the hypercubes and upload them to OMERO server. The example implementation is the ‘DefaultImageProcessor’ in the ‘omero_data_transfer.default_image_processor’ module.

For the convenience of developers, we created the omero_ide Docker which provides JetBrains’ PyCharm ^31^ IDE (Integrated Development Environment) and Codium ^32^, the Open Source software binaries of VSCode ^33^. The container runs an OpenSSH ^34^ server that enables users to establish an X11 ^35^ SSH connection so that the IDE GUI can be displayed, as if the IDE is running on the host system. For Linux and Mac OS users, the connection can be established simply by entering the standard `ssh -X jovyan@127.0.0.1 -p 2222` in the command terminal. For Windows users, an *X Server* application must be installed such as MobaXTerm ^36^ or XMing ^37^.

### Use case 4: updating the OMERO Python library dependency

Like all software packages, maintainability of the toolkit is vital to achieve sustained, long term use. Since the OMERO software itself is regularly updated and the OMERO libraries are sensitive to parity between versions, it is essential to equip the toolkit with a convenient mechanism for updating the constituent versions.

In order to update the core OMERO Python library, the omero_base image Dockerfile contains two static variables: ‘OMERO_VERSION’ and ‘ZEROC_ICE_PACKAGE’. If necessary, these variables can be modified to meet the requirements of the target OMERO server version. The only requirement is that the relevant ‘omero-py’ library ^14^ is available in the Python Package Index (PyPI) repository ^15^. After updating these version variables, then the Docker images need to be rebuilt as described in their README. Fortunately, the PyOmeroUpload toolkit is resilient to differences between versions of the core OMERO client library and target server; for example, v5.6.2_2.0.0 – which depends on version 5.6.2 of the OMERO client library – has been tested and found to be compatible with target servers running OMERO 5.4.10. This feature mitigates the need for regular updates in the Docker images to keep pace with OMERO releases, and allows one uploader Docker container to interoperate with multiple different servers running various versions of OMERO.

### Results from a real example

The PyOmeroUpload toolkit was used to upload two datasets to the public OMERO demonstration server. The original microscopy data in each dataset comprised around 90,000 individual 512 × 512 pixel, covering 25 microscope positions, 3 channels, 5 z-sections and 240 timepoints. This amounted to approximately 30GB per dataset. The data in each dataset was structured into one directory per microscope position, containing individual files that adhered to a file naming convention specifying the channel, z-section and timepoint of each image, with metadata residing at the top directory level in two semi-structured text files. The toolkit was executed using the default included metadata parser and image processor classes as described in the *Operation* section above. The project containing these datasets is published on the University of Edinburgh School of Biology public OMERO server at https://publicomero.bio.ed.ac.uk/webclient/?show=project-55. After uploading, each dataset contained 25 hypercube images corresponding to each microscope position, with the individual components of each hypercube accessible in the OMERO server full viewer.

## Discussion

Modern research relies on various data types and management techniques, so it is implausible to conceive of one software platform that can cater for the needs of all research groups even within a moderate-sized Department. Specialized resources and tools such as OMERO, which is dedicated only to microscopy data, and Jupyter notebooks for data analysis, are key to providing satisfactory user experience and unlocking maximum value from using a data repository. However, the diverse systems must interoperate such that relationships between different datasets are captured and existing metadata are reused. Minimizing the “human” factor in data deposition and automation of the process assures high quality data and improves the productivity of experimental researchers. Our work seeks to facilitate programmatic access to data resources, as a key contribution to the integrated and sustainable research data management envisaged by the FAIR principles ^38^.

Our expected users are biological or biomedical researchers who apply, and perhaps develop, automated microscopy and analysis to perform increasingly data-rich studies. They necessarily prioritize research innovation and data generation, rather than underpinning software development. We therefore address the interaction of this user group with the specialized resources that add value to data for them.

The many benefits of the OMERO ecosystem are noted above. Development of the toolkit was driven by a few, specific barriers for researchers wishing to deposit their data conveniently into OMERO: namely, dependencies on OpenSSL system libraries and Java security certificates; compatibility between different versions of client libraries and server installations; and the resulting deleterious impact on the portability of solutions built to harness the power and adaptability of the Python OMERO API. Many of these issues have been addressed with the recent release of omero-py 5.6 ^14^, especially the difficulties around installation of Python OMERO libraries on Microsoft Windows OS, which are now largely resolved since the upgrade to Python 3 and migration to PyPi. Prior to this release, the OMEROConnect Docker images provided a viable and convenient method for Windows users to utilize the OMERO Python library. While the OMERO ecosystem provides good user experience for manual interaction with microscopy data, we tried to address some of the issues with automated or programmatic access to the repository from a Python environment.

One potential feature that would benefit OMERO is the implementation of a full featured RESTful ^39^ API. Using standard HTTP methods to upload and retrieve (meta)data in an OMERO server through JSON payloads and multipart file attachments would completely remove the dependency on a bespoke client library (other than one for handling HTTP requests), thus maximizing interoperability. However, it must be acknowledged that many of the file formats supported by OMERO via the Bio-Formats library are complex, with multiple constituents and particular conversion sequences that are performed on the client side rather than on the server. OMERO does expose a JSON API which provides some CRUD (Create, Read, Update, Delete) operations but it does not mimic all the functions available in the client library.

As an effective compromise between flexibility and interoperability, the RESTful API could make use of an open, exchangeable image format that would streamline the image data ingest process; a perfect candidate for this standardisation would be the OME-TIFF format. The advantages with this format are that image planes constituting a 5D image can be stored within one multi-page TIFF file, and OME-XML metadata blocks are embedded in each TIFF file’s header ^40^, so it is extremely portable. The format also supports the generation of pyramidal levels from large resolution planes ^41^, allowing whole slide images or very large acquisitions to be handled efficiently in OMERO.

Utilisation of the OME-TIFF format could present an alternative approach to the current implementation of PyOmeroUpload, which leverages raw pixel format, and in principle PyOmeroUpload could convert image directory structures into OME-TIFF using the existing libraries ^42, 43^. However, uploading image data in its original format, rather than in raw pixel format, requires use of the OMERO CLI tool. We found this method is less portable because it depends on additional binaries being installed and we had difficulty making it work in a Jupyter Notebook environment. Ideally, the benefits of the OME-TIFF format for direct upload to an OMERO server could be maximised through a native Python image import implementation or the RESTful API.

In the future, PyOmeroUpload could be combined with additional image processing implementations that could make use of machine or deep learning libraries which would pre-process the image data, performing tasks such as denoising, segmentation and feature detection. The results could be applied as ROIs to the images in OMERO, further enriching attached metadata.

## Summary

The PyOmeroUpload toolkit assists users of OMERO with the tasks of uploading, annotating and sharing their (meta)data using programmatic access from a python environment. It provides an extensible framework for automating a data deposition workflow by allowing specification of metadata extraction and image processing modules, while insulating the user from the lower-level interactions and exposing a simpler API for typical data sharing tasks.

## Data availability

The sample data utilized in the upload demonstration operations are maintained in the SynthSys GitHub repository at https://github.com/SynthSys/omero_connect_demo/releases/tag/v1.0.0.

Data relating to the referenced Granados (2018) paper are published at https://publicomero.bio.ed.ac.uk/webclient/?show=project-55

## Software availability

### PyOmeroUpload Toolkit

1. Docker images are available from: https://hub.docker.com/r/biordm/omero-connect
2. Source code is available from: https://github.com/SynthSys/pyOmeroUpload
3. Release of source code at time of publication is available from: https://github.com/SynthSys/pyOmeroUpload/releases/tag/v5.6.2_2.2.0
4. Archived source code at time of publication: https://doi.org/10.5281/zenodo.3982334 ^44^
5. Licence: MIT

### OMEROConnect Docker Images

1. Docker images are available from: https://hub.docker.com/r/biordm/omero-connect
2. Source code is available from: https://github.com/SynthSys/OMEROConnect
3. Release of source code at time of publication is available from: https://github.com/SynthSys/OMEROConnect/releases/tag/v5.6.2_2.2.0
4. Archived source code at time of publication: https://doi.org/10.5281/zenodo.3982311 ^45^
5. Licence: MIT

### OMEROConnect Demo Notebooks

1. Source code is available from: https://github.com/SynthSys/omero_connect_demo
2. Release of source code at time of publication is available from: https://github.com/SynthSys/omero_connect_demo/releases/tag/v1.0.0
3. Archived source code at time of publication: https://doi.org/10.5281/zenodo.3746514 ^46^
4. Licence: MIT
