# Supplementary material for: PyOmeroUpload: A Python toolkit for uploading images and metadata to OMERO
Source: Wellcome Open Res. 2020 Aug 26;5:96. Originally published 2020 May 18. [Version 2] doi: 10.12688/wellcomeopenres.15853.2 (PMC7388197; doi:10.12688/wellcomeopenres.15853.2)
Supplement: Peer review history

# Peer review: Reviewer response for version 2

**DOI:** 10.21956/wellcomeopenres.17849.r40177

**Published:** 2020-08-27

**Authors:** Anatole Chessel

**Affiliations:** 1 LOB, École polytechnique, Institut Polytechnique de Paris, Paris, France

No further comments.

Are the conclusions about the tool and its performance adequately supported by the findings presented in the article?

Yes

Is the rationale for developing the new software tool clearly explained?

Yes

Is the description of the software tool technically sound?

Yes

Are sufficient details of the code, methods and analysis (if applicable) provided to allow replication of the software development and its use by others?

Yes

Is sufficient information provided to allow interpretation of the expected output datasets and any results generated using the tool?

Yes

Reviewer Expertise:

Bioimage informatics, biological data science, computational biology

I confirm that I have read this submission and believe that I have an appropriate level of expertise to confirm that it is of an acceptable scientific standard.

# Peer review: Reviewer response for version 1

**DOI:** 10.21956/wellcomeopenres.17390.r39530

**Published:** 2020-07-28

**Authors:** Anatole Chessel

**Affiliations:** 1 LOB, École polytechnique, Institut Polytechnique de Paris, Paris, France

Hay *et al.* propose a toolkit in python meant to help with the loading of imaging data and metadata into an OMERO instance. They provide easy to use docker containers all the way to jupyter notebooks exemplifying the use of their tool on a demo server.

 While the idea of FAIR open data is increasingly getting traction across the scientific community, in actual practice many issues, sometime seemingly low level, can make the actual sharing, or even just local management, of complex data surprisingly hard. The PyOmeroUpload toolkit, which interact with OMERO, arguably the most widely used client-server software for microscopy image management, aims at easing one key step of data management workflow, actually getting data in the data management system. Great care has obviously been put in making an easy to use and easy to maintain system and the paper is well written and very didactic, and one can only hope that it will be used in practice and ease the life of many researchers worldwide.

 A few minor comments/questions:

- More advanced examples, including a few line of standard ‘*Acq.txt’ and ‘*log.txt’, or the predefined regular expression, would have helped in showing what is available out of the box and what would need additional work to implement.
- Increasingly acquisition are becoming so large as to need specialized pyramidal format. A few of those are now available in OMERO, how compatible would PyOmeroUpload be with them?
- Some advanced options of OMERO import include linking the data instead of copying it, to avoid data duplication. Would they be easy to include in PyOmeroUpload?
- As noted by the authors, the addition of ROIs would be an interesting next step, either via 'on the fly' analysis or the reading of precomputed rois files. The format of those is even less standard that images unfortunately...

Are the conclusions about the tool and its performance adequately supported by the findings presented in the article?

Yes

Is the rationale for developing the new software tool clearly explained?

Yes

Is the description of the software tool technically sound?

Yes

Are sufficient details of the code, methods and analysis (if applicable) provided to allow replication of the software development and its use by others?

Yes

Is sufficient information provided to allow interpretation of the expected output datasets and any results generated using the tool?

Yes

Reviewer Expertise:

Bioimage informatics, biological data science, computational biology

I confirm that I have read this submission and believe that I have an appropriate level of expertise to confirm that it is of an acceptable scientific standard.

# Response

**Published:** 2020-08-14

**Authors:** Johnny Hay

Thank you for your constructive and positive review, our detailed responses are listed below : 

- More advanced examples, including a few line of standard ‘*Acq.txt’ and ‘*log.txt’, or the predefined regular expression, would have helped in showing what is available out of the box and what would need additional work to implement.
  - We have added an excerpt of the example log.txt file in the 'Use Case 1' section, and we hope that users can refer to the 'omero_connect_demo' example data repository for further details. The provided parser supports all the forms of annotations currently available in OMERO. 

 

- Increasingly acquisition are becoming so large as to need specialized pyramidal format. A few of those are now available in OMERO, how compatible would PyOmeroUpload be with them?
  - This is an excellent point: supporting large resolution pyramidal formats would be a very useful feature for PyOmeroUpload. We added discussion of using OME-TIFF format as an alternative approach in the discussion section. 

- Some advanced options of OMERO import include linking the data instead of copying it, to avoid data duplication. Would they be easy to include in PyOmeroUpload?
  - Due to the hierarchical many-to-many data structure in OMERO, it is possible and indeed preferable to link multiple different parent containers with the same child images rather than copy these child elements, which results in inefficiencies of duplication.The challenges to implementing this feature are primarily related to defining ‘uniqueness’ in uploaded data: that is, which attributes of an entity in OMERO constitute its unique ID? In the case of uploaded images, PyOmeroUpload could of course run a preliminary scan of all the images owned by the active user to compare original filenames or titles, and if the filename of the image to be uploaded already exists on the server. However, that is very use case specific i.e. depends on naming conventions, tags used etc. so such feature enhancement in PyOmeroUpload would require careful consideration, although technically it is entirely feasible

- As noted by the authors, the addition of ROIs would be an interesting next step, either via 'on the fly' analysis or the reading of precomputed rois files.
  - The most suitable candidate format for standardising and persisting OMERO ROI annotations is OME-XML. At present the main mechanism for importing and exporting ROIs that we found is the OME ROI tool (https://github.com/glencoesoftware/ome-omero-roitool), which is a Java implementation for parsing OME-XML. To integrate this tool, it would be necessary to invoke the Java application as a process from the PyOmeroUpload toolkit. Alternatively, it may be possible to either embed the OME-XML in an OME-TIFF image file header, or to attach the ROIs as separate OME-XML file annotations linked with the image. However, it is not immediately clear how easily these file annotations could then be viewed in the OMERO UI. It would certainly be an interesting feature to explore, especially to enable easier sharing of image analyses. 

# Peer review: Reviewer response for version 1

**DOI:** 10.21956/wellcomeopenres.17390.r38747

**Published:** 2020-06-25

**Authors:** Jason R Swedlow, Sébastien Besson, Josh Moore, Jean-Marie Burel

**Affiliations:** 1 Centre for Gene Regulation and Expression, School of Life Sciences, University of Dundee, Dundee, UK

**Summary**

 This manuscript describes an extension of OMERO, the open source data management system built and released by OME. PyOmeroUpload is a series of Python tools, packaged in a Docker container, that aims to simplify the upload and import of imaging datasets from the command line. Overall, the paper is clearly written and PyOmeroUpload is a great example of how the wider community can extend open source infrastructure and tools to meet the needs of imaging data handling, curation and publication. The default pipeline includes tools that convert imaging data stored in a custom internal file format and publish it into a multi-dimensional representation enriched with tags and key/value pairs. A few core metadata concepts are missing from the generated output like physical pixel sizes as well as channel metadata like wavelengths however, these can be added in later iterations of the tool. The authors have included a public data set generated by the software as a proof-of-concept and this is very useful for assessing output.

 Minor Points. These all reflect impressions of the PyOmeroUpload itself. The manuscript is quite clear and easy to comprehend.

1. PyOmeroUpload uses the OMERO API to create OMERO multi-dimensional images. Has the alternate approach of converting the original files into an open exchangeable format like OME-TIFF been discussed as part of the design (cf http://blog.openmicroscopy.org/community/file-formats/2019/06/25/formats/, https://forum.image.sc/t/converting-whole-slide-images-to-ome-tiff-a-new-workflow/32110)? 
2. In terms of curated metadata, while PyOmeroUpload offers some flexibility on types of annotations the user can access, the public proof of concept hosted on the Univ of Edinburgh server uses tags as the primary mechanism to capture and search annotations. An example using key/value annotations might be also useful.  We suggest this-- and it is only a suggestion--  only because K-Vs have proven to be extremely powerful for annotation and search in our work on IDR ( https://www.nature.com/articles/nmeth.4326).
3. Thinking about ease of use and adoption of the tool, it seems that the user needs to define a valid file *log.txt and a file *acq.txt. Ideally this should be optional to make the application more generic. The authors might consider making these yml files.   
4. For the purposes of ****promoting PyOmeroUpload and capturing provenance, the data it imports could include an annotation that refers to the tool used for import. Has there been some consideration of including the name and version of the tool and maybe the DOI of the paper as systematic tags and or key/value pairs?

Are the conclusions about the tool and its performance adequately supported by the findings presented in the article?

Yes

Is the rationale for developing the new software tool clearly explained?

Yes

Is the description of the software tool technically sound?

Yes

Are sufficient details of the code, methods and analysis (if applicable) provided to allow replication of the software development and its use by others?

Yes

Is sufficient information provided to allow interpretation of the expected output datasets and any results generated using the tool?

Yes

Reviewer Expertise:

cell biology, cell cycle, imaging, image informatics

We confirm that we have read this submission and believe that we have an appropriate level of expertise to confirm that it is of an acceptable scientific standard.

# Response

**Published:** 2020-08-14

**Authors:** Johnny Hay

Thank you for your constructive and positive review, we are pleased to know that the main OMERO authors find our toolkit to be a useful extension. Our detailed responses are listed below: 

- PyOmeroUpload uses the OMERO API to create OMERO multi-dimensional images. Has the alternate approach of converting the original files into an open exchangeable format like OME-TIFF been discussed as part of the design. 
  - This is an excellent idea, and we have added a review of the benefits of converting the original files into OME-TIFF format in the discussion section. However, we had difficulty uploading images directly in the original format using the existing API due to the dependency on additional CLI binaries.  

 

- In terms of curated metadata, while PyOmeroUpload offers some flexibility on types of annotations the user can access, the public proof of concept hosted on the Univ of Edinburgh server uses tags as the primary mechanism to capture and search annotations. An example using key/value annotations might be also useful.   
  - The KVP annotation is indeed a very powerful feature. PyOmeroUpload toolkit does in fact already provide support for automatically extracting KVPs. An excerpt of the relevant experimental log file has been added to the 'Use Case 1' section along with a screenshot of the KVPs as they appear on the OMERO UI.

 

- Thinking about ease of use and adoption of the tool, it seems that the user needs to define a valid file *log.txt and a file *acq.txt. Ideally this should be optional to make the application more generic. The authors might consider making these yml files.     
  - This suggestion is an elegant solution to address the dependency on the *log.txt and *acq.txt in the default metadata parser. In the latest release of PyOmeroUpload, we have introduced another CLI parameter '-x' that allows users to specify whether the metadata extraction task should be skipped. Additionally, if the user forgets to specify this argument, the application applies enhanced logic to detect whether the file(s) are missing and report back to the user if so, but continues with the upload process regardless. 
    The semi-structured metadata format (the log and acq files) are “biologist” friendly, and as such they can be easily understood/edited by users. Based on our experience, yml files are perceived as more complex and daunting, although they would be far preferable in terms of programmatic interoperability and metadata structure. 

 

- For the purposes of promoting PyOmeroUpload and capturing provenance, the data it imports could include an annotation that refers to the tool used for import.  
  - Another great suggestion, and we have added these Key/Value pairs to uploads by default: ['Uploaded With', 'pyOmeroUpload x.x.x'] and ['PyOmeroUpload', ' https://github.com/SynthSys/pyOmeroUpload']. These metadata elements can be disabled by the user through another CLI parameter if desired. 
